# A central role for p38 MAPK in the early transcriptional response to stress

**DOI:** 10.1186/1741-7007-8-47

**Published:** 2010-04-24

**Authors:** Alan J Whitmarsh

**Affiliations:** 1 Faculty of Life Sciences, University of Manchester, Michael Smith Building, Oxford Road, Manchester M13 9PT, UK

## Abstract

The mitogen-activated protein kinase p38 (p38 MAPK) is activated by a number of stresses. A recent study in *BMC Genomics *has uncovered the early transcriptional responses to three types of stress and has demonstrated a central role for p38 MAPK in mediating these responses.

See research article http://www.biomedcentral.com/1471-2164/11/144

## Commentary

When cells are subjected to stress, such as osmotic stress, heat shock or infection, a number of intracellular signaling pathways are activated, leading to a coordinated cellular response. This may include the cessation of the cell cycle, commitment to apoptosis, the activation of DNA-repair pathways, regulation of protein translation, and the initiation of immune responses. A large body of evidence over the past 15 years has demonstrated that the p38 mitogen-activated protein kinase (MAPK) pathway is an important mediator of these stress responses [1,2]. What is less clear is how the p38 MAPK pathway regulates an appropriate program of gene expression to allow cells to respond to particular stresses. A study published recently in *BMC Genomics *by Ferreiro *et al*. [3] indicates a pivotal role of the p38 pathway in the early stress-induced transcriptional response to distinct stress stimuli.

## Stress and the p38 pathway

The p38 pathway, like other MAPK pathways, features a cascade of protein kinases, culminating in the phosphorylation of p38 MAPK on specific threonine and tyrosine residues within its activation loop by the dual-specificity MAPK kinases MKK3 and MKK6, thereby leading to its activation [1,2]. There are four genes encoding p38 family members in mammals, with the p38α isoform being the most abundant in the majority of cell types [2]. Various mouse models have demonstrated that p38α is important for cytokine production, inflammatory responses and controlling cell proliferation [2]. A number of direct substrates of p38α have been identified, including the transcription factors ATF2 (a bZIP family transcription factor with diverse roles in development, cell growth and survival), MEF2 (important for cell differentiation and organogenesis), DDIT3 (produced in response to DNA damage), and the tumor suppressor p53 [4].

Studies in yeast and nematodes, where the p38 pathway is conserved, have shed some light on how it regulates the transcriptional response to stress. The *Saccharomyces cerevisiae *p38 homolog Hog1 is primarily activated by osmotic stress and mediates a specific program of gene expression to combat and adapt to high osmolarity [5]. This is, in part, mediated by Hog1 regulating a number of transcription factors that target the promoters of osmotic-stress responsive genes and, in some instances, also being recruited itself to promoter-bound transcriptional complexes and directly regulating their assembly and activity [5]. In the nematode *Caenorhabditis elegans*, genetic experiments have established a role for p38 in innate immunity and transcription profiling has demonstrated that p38 is required for upregulation of pathogen-response genes [6]. In mammals there have only been a few studies on the genome-wide transcriptional response to stress mediated by p38 and these have focused on longer-term transcriptional changes rather than the immediate response (see, for example, [7,8]).

## p38-dependent transcriptional responses

To uncover the immediate p38-dependent transcriptional response to stress, Ferreiro *et al*. [3] used two approaches: mouse embryonic fibroblasts (MEFs) genetically lacking p38α and treatment with an inhibitor of both p38α and the less abundant p38β isoform. Largely similar results were obtained with both approaches, although p38 dependency using the inhibitor appeared greater than in the p38α-knockout MEFs, possibly because these cells can adapt during mouse development to use other pathways, or because some genes are dependent on p38β. Three distinct stress stimuli were chosen: tumor necrosis factor-α (TNFα), a pro-inflammatory cytokine released at the sites of cell damage; high osmolarity; and the protein-synthesis inhibitor anisomycin. These stimuli give distinct p38α-activation profiles, with TNFα causing a rapid but transient increase in activity whereas osmotic stress and anisomycin cause more prolonged activation [3]. Gene-expression analysis following short exposures to the stresses (45 minutes for anisomycin and TNFα, 2 hours for osmotic stress) showed that between 60 and 88% of the upregulated genes were dependent on p38α (defined by a greater than 50% loss of expression in either the knockout or drug-treated cells), and that the majority of the genes were regulated in a stimulus-specific manner, with only a small core set of genes being upregulated by all three stimuli [3]. While the study mainly focuses on the analysis of upregulated genes, a handful of genes were downregulated, and this response was also highly dependent on p38. The findings confirm a central role for p38 in the transcriptional response to stress and suggest that the p38 pathway collaborates with distinct stimulus-specific pathways and transcription factors.

**Figure 1:**
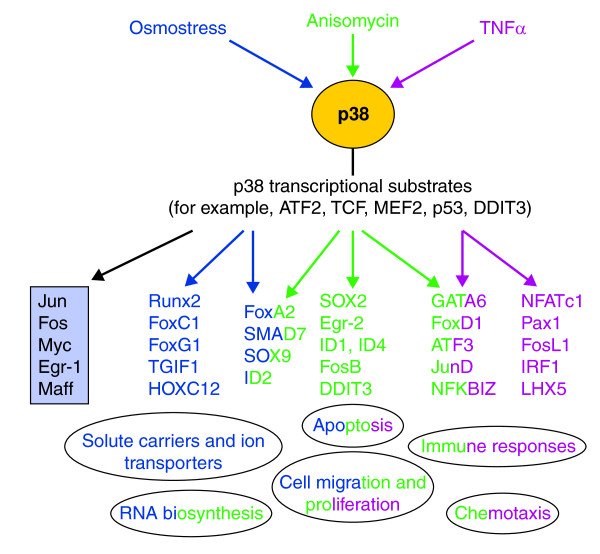
**p38 MAPK mediates the transcriptional response to stress**. The p38 MAPK pathway is activated by stresses and leads to the phosphorylation of a number of transcriptional regulators that can orchestrate a program of gene expression, including the upregulation of many transcription-factor genes. The transcription-factor genes listed in the light-blue shaded box were upregulated by all the stresses to some extent. The other transcription-factor genes listed are color coded and are examples of genes that are upregulated by individual stresses or by two of the stresses (dark blue, osmotic stress; green, anisomycin; purple, TNFα). Functional categories of genes that were enriched are depicted at the bottom of the figure.

Which genes are upregulated by the stimuli and how do they function in the cellular response to stress? To begin to address this important question, Ferreiro *et al*. [3] analyzed the regulated genes on the basis of Gene Ontology (GO) categories and looked for the selective enrichment of genes associated with particular cellular components, molecular functions and biological processes. In addition, they performed Ingenuity Pathway Analysis (IPA), which makes use of a bibliographic database to enable the identification of relevant gene networks and their associated functions. As might be predicted, the gene set upregulated by TNFα was enriched for genes associated with the immune response and the stress response, and included genes for several chemokines and cytokines. Anisomycin treatment also upregulated chemokine and cytokine genes, but to a lesser extent than with TNFα, suggesting a partial overlap in the responses to the two stimuli (Figure 1). The genes that were upregulated by osmotic stress were enriched for those associated with RNA biosynthesis pathways, cell migration, apoptosis and the regulation of proliferation, and fitted with a gene network associated with the control of cell cycle and posttranslational modification. Again, there was some overlap between responses as the anisomycin-upregulated genes were also enriched for components of RNA biosynthesis pathways, whereas all the stresses showed some enrichment for regulators of cell migration, proliferation and apoptosis (Figure 1).

To obtain a fuller picture of how changes in transcription direct the cellular response to stress, Ferreiro *et al*. [3] analyzed the genes upregulated by osmotic stress over a longer time-scale. They found that the gene-expression profiles changed significantly over time and that the dependence on p38 decreased. Although it is possible that these effects may be specific to osmotic stress, studies in different cell types at later time points following stimulation with TNFα indicate that, at this stage, fewer genes are p38 dependent compared with the immediate TNFα response measured by Ferreiro *et al*. and there is little overlap in the pool of genes upregulated [3,7,8]. This further underscores the importance of p38 for the early transcriptional responses to stress.

The temporal changes in the transcriptional profiles in response to osmotic stress can be linked to specific cellular responses. For example, short exposures to osmotic stress (45 minutes) led to the upregulation of solute carriers and ion transporters to allow cells to rapidly counteract the differences in osmotic pressure between the inside of the cell and the environment. Longer exposures (2 hours) results in the upregulation of genes encoding transcription factors and those involved in ribosome biogenesis, indicating a requirement for new protein synthesis, whereas 8 hours of exposure promotes the expression of genes involved in regulating cell survival and cell growth as well as cytokine and chemokine genes, suggesting a strong defense response.

## Upregulation of transcription-factor genes by p38

One of the significant findings of the study was that the most highly enriched functional grouping across all three stresses was for genes encoding transcription factors (Figure 1). This was also evident for the subset of genes that are upregulated by all the stresses, with 5 out of 30 being transcription factors. These include genes encoding the ubiquitously expressed AP-1 family members Jun and Fos, as well as Myc, Egr-1 and Maff (Figure 1). Some of these have previously been reported to be p38 pathway targets [4]. For example, p38 may regulate *jun *transcription via MEF2 and ATF2, two direct substrates of p38, whereas *fos *and *egr-1 *can be regulated by other p38 substrates, such as ternary complex factors (TCFs) [1,2,4]. The enrichment for transcription-factor genes indicates that cells require an extensive program of new gene expression for long-term adaptation to stress.

For the future it will be important to determine how the p38-mediated gene-expression profiles differ between cell types and in response to other types of stress. The prediction, based on the study by Ferreiro *et al*. [3], would be that most of the genes will be regulated in a stress-specific manner but there will be a core set of key genes upregulated by all the stresses. Similarly, it is likely that there will be differences in gene expression between cell types in response to a specific stress, but that a core set of genes will be required for all cells to adapt to that particular stress. The study by Ferreiro *et al*. [3] establishes p38 as an essential regulator of the early transcriptional response to stress and is an important starting point for uncovering the complexities of the stress response and how it changes during development and in disease. It also provides a platform on which to probe in more detail the transcriptional network controlled by the p38 pathway. This could include high-throughput protein kinase assays to identify the full complement of transcriptional substrates of p38, combined with integrating gene-expression data with chromatin immunoprecipitation (ChIP) analysis of p38 and p38 substrates. This would allow predictive models of the p38-regulated transcriptional network to be generated, which could potentially lead to the identification of therapeutic targets within the network that could be exploited to treat immune disorders and cancers.

## Abbreviations

ChIP: chromatin immunoprecipitation; GO: Gene Ontology; IPA: Ingenuity Pathway Analysis; MAPK: mitogen-activated protein kinase; MEFs: mouse embryonic fibroblasts; MKK: MAPK kinase; TNFα: tumor necrosis factor-α.
